# Supplementary material for: Learning curve and surgical outcome of robotic assisted colorectal surgery with ERAS program
Source: Sci Rep. 2022 Nov 29;12:20566. doi: 10.1038/s41598-022-24665-w (PMC9709162; doi:10.1038/s41598-022-24665-w)
Supplement: Supplementary file 2 — Supplementary Information 2. [file 41598_2022_24665_MOESM2_ESM.zip › RAL Raw data-Ñ[▒K/069/OP N_ñw╝╨░O▒Kñσ.pdf]

W55 / W55

臺中榮民總醫院
手術室護理記錄

[redacted]

基本資料

- 室別： 開刀房-17室，手術科別： CRS，手術類別： 常規，日期： 2021/03/31
- 進入時間： 1550 ； 麻醉開始時間： 1600 ； Time Out時間： 1725 ； 劃刀時間： 1726 ； 手術結束時間： 0120 送出時間： 0140 ；
- 麻醉方式： GE

  診斷： Malignant tumor of anal canal and anus. APR

  手術名稱： 1.Robot Assisted Low Anterior Resection
  2.Incisional biospy

  手術局部用藥： NIL

  術前預防性抗生素：
  - 抗生素名稱一： Veterin 1GM IVD由麻醉護理師給予
  - 時間： 1720
  - 上次簽章: 李郁萱

  ，追加預防性抗生素：
  - 抗生素名稱一： Veterin 1GM IVD由麻醉護理師給予
  - 時間： 2120
  - 上次簽章: 李郁萱
- 手術醫師： 林俊余，蔡岳錤，楊曜榮，林陳彥，陳智翔，王郁棋
- 麻醉醫師： VS 齊光彪
-
  - 刷手護士A ： 廖子晴，時間： 1550，刷手護士B ： 蘇敬涵，時間： 1550，接替刷手一： 邱儷綵，時間： 1930，接替刷手二： 廖子晴，時間： 2200
  - 流動護士A ： 李郁萱，時間： 1550，接替流動一： 陳心慈，時間： 2300
- 更多接替及流動護士： 接替刷手三： 張玉梅，時間： 0000

皮膚狀況

A-V Shunt： 無

術前： 完整

記錄者： 上次簽章: 李郁萱

術後： 完整

記錄者： 陳心慈

手術準備

手術臥式

-
  - 手術臥式一： 仰臥
  - 使用附件： 溫毯 38 ℃，頭圈
  - 皮膚準備： 其它： 2% CHLORHEXIDINE
  - 電燒： 使用電燒: 電極片位置： 右小腿，記錄者： 上次簽章: 李郁萱
-
  - 手術臥式二： 垂頭仰臥式
  - 使用附件： 溫毯 38 ℃，頭圈，腳架，其它： LATERAL SUPPORT
  - 皮膚準備： 10%Aq Betadine，Hibiscrub，其它： 2% CHLORHEXIDINE
  - 電燒： 使用電燒: 電極片位置： 右大腿，記錄者： 上次簽章: 李郁萱

止血帶T： 無

植入物

- 植入物名稱/號碼/數量/部位： DAVINCI XI 45mmXmm* 1 / COLON

，記錄者： 陳心慈

標本送檢

- 細菌培養 無
- 細胞學 無
- 冰凍切片 無
- 病理標本
  組織名稱一：L't GROIN SOFT TISSUE
  ,
  組織名稱二：IMA ROOT
  ,
  組織名稱三：RECTUM
- 截肢 無

標本交付

-
  - 標本： 1 袋
  - 標本： 0 盒
  - 標本： 0 瓶
  - 細胞學： 0 管
  - Culture Tube： 0 管
  - 被交付醫師： VS 蔡岳錤
  -
    - 記錄者： 上次簽章: 李郁萱
    - 時間： 1900
-
  - 標本： 2 袋
  - 標本： 0 盒
  - 標本： 0 瓶
  - 細胞學： 0 管
  - Culture Tube： 0 管
  - 被交付醫師： R 陳智翔
  -
    - 記錄者： 陳心慈
    - 時間： 0140

傷口術後敷料

傷口敷料： 傷口敷料： 無

- 請於圖上標示部位：

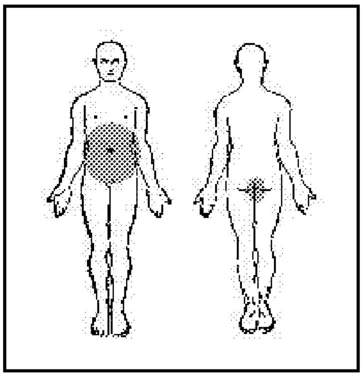

- 傷口敷料： 腹部：軟性敷料
  會陰部：GAUZE

導管

導管	天數	導管資訊	插入日期	插入時間	更換日期	拔除日期	拔除時間	拔除原因	預計拔除/更換	預計拔除/更換日期
Foley(1)	2	號碼:16 Fr.; 部位:膀胱;	2021/03/31	1700						
A-line (1)	2	號碼:20 G; 部位:左手;	2021/03/31	1630						
Epidural PCA(1)	2	部位:硬脊膜;	2021/03/31	1615						
Double		號碼:8 Fr.; 深度:15cm; 部								

Lumen CVC(1)	2	位:jugular (右); 象限:RU;	2021/03/31	1640						
Penrose Drain(1)	1	號碼:12 mm.; 部位:會陰部;	2021/04/01	0100						
Chemo Port(1)	213	部位:左鎖骨下;	2020/09/01	0800						

用物計數

- 手術中出血量： 少量 c.c.
- 手術中尿量： 300 c.c.
-
  - 其它： 無

用物計數：

- 部位一： INGUINAL，1.器械： 正確，2.縫針： 正確，3.刀片： 正確，4.紗布： 正確，5.腹部墊： 未使用，6.紗球： 未使用，7.PEANUTS： 未使用，8.紗條： 未使用，9.絲帶： 未使用，10.針頭： 未使用，11.Vessel loop： 未使用，12.Boots： 未使用，13.Jelco： 未使用，14.棉條： 未使用，
  - 第一次：
    - 刷手護士 ： 廖子晴
    - 流動護士： 上次簽章: 李郁萱
    - 時間： 1840

  ,
  - 第二次：
    - 刷手護士 ： 廖子晴
    - 流動護士： 上次簽章: 李郁萱
    - 時間： 1845

  ,
  - 第三次：
    - 刷手護士 ： 廖子晴
    - 流動護士： 上次簽章: 李郁萱
    - 時間： 1850
- 部位二： ABDOMEN，1.器械： 正確，2.縫針： 正確，3.刀片： 正確，4.紗布： 正確，5.腹部墊： 未使用，6.紗球： 未使用，7.PEANUTS： 未使用，8.紗條： 未使用，9.絲帶： 未使用，10.針頭： 未使用，11.Vessel loop： 未使用，12.Boots： 未使用，13.Jelco： 未使用，14.棉條： 未使用，
  - 第一次：
    - 刷手護士 ： 廖子晴
    - 流動護士： 陳心慈
    - 時間： 2355

  ,
  - 第二次：
    - 刷手護士 ： 張玉梅
    - 流動護士： 陳心慈
    - 時間： 0030

  ,
  - 第三次：
    - 刷手護士 ： 張玉梅
    - 流動護士： 陳心慈
    - 時間： 0040
-
  - 第一次：
    - 流動護士：

  ,
  - 第二次：
    - 流動護士：

  ,
  - 第三次：
    - 流動護士：

備註

備註： DAVICI 0度 SCOPE NO.1

- 記錄者： 上次簽章: 李郁萱
- 時間： 2100

填寫護理人員 陳心慈

正式摘要

	W55 / W55

doctorRank:B
